# Eye-Movement Training Results in Changes in qEEG and NIH Stroke Scale in Subjects Suffering from Acute Middle Cerebral Artery Ischemic Stroke: A Randomized Control Trial

**DOI:** 10.3389/fneur.2016.00003

**Published:** 2016-01-22

**Authors:** Frederick Robert Carrick, Elena Oggero, Guido Pagnacco, Cameron H. G. Wright, Calixto Machado, Genco Estrada, Alejandro Pando, Juan C. Cossio, Carlos Beltrán

**Affiliations:** ^1^Neurology, Carrick Institute, Cape Canaveral, FL, USA; ^2^Global Clinical Scholars Research Training Program (GCSRT), Harvard Medical School, Boston, MA, USA; ^3^Institute of Neurology and Neurosurgery, Havana, Cuba; ^4^Bedfordshire Centre for Mental Health Research, University of Cambridge, Cambridge, UK; ^5^Electrical and Computer Engineering, University of Wyoming, Laramie, WY, USA

**Keywords:** ischemic stroke, qEEG, NIHSS scores, eye moment therapy, stroke rehabilitation, saccades

## Abstract

**Context:**

Eye-movement training (EMT) can induce altered brain activation and change the functionality of saccades with changes of the brain in general.

**Objective:**

To determine if EMT would result in changes in quantitative electroencephalogram (qEEG) and NIH Stroke Scale (NIHSS) in patients suffering from acute middle cerebral artery (MCA) infarction. Our hypothesis is that there would be positive changes in qEEG and NIHSS after EMT in patients suffering from acute MCA ischemic stroke.

**Design:**

Double-blind randomized controlled trial.

**Setting and participants:**

Thirty-four subjects with acute MCA ischemic stroke treated at university affiliated hospital intensive care unit.

**Interventions:**

Subjects were randomized into a “control” group treated only with aspirin (125 mg/day) and a “treatment” group treated with aspirin (125 mg/day) and a subject-specific EMT.

**Main outcome measures:**

Delta–alpha ratio, power ratio index, and the brain symmetry index calculated by qEEG and NIHSS.

**Results:**

There was strong statistical and substantive significant improvement in all outcome measures for the group of stroke patients undergoing EMT. Such improvement was not observed for the “control” group, and there were no adverse effects.

**Conclusion:**

The addition of EMT to a MCA ischemic stroke treatment paradigm has demonstrated statistically significant changes in outcome measures and is a low cost, safe, and effective complement to standard treatment.

## Introduction

Stroke is one of the leading causes of death in the United States and is a major cause of adult disability; although from 2001 to 2011 the relative rate of stroke death fell by 35.1% and the actual number of stroke deaths declined by 21.2%, the number of persons suffering from a stroke is still significant (≈795,000 each year in the United States alone) and its consequences are serious (in 2011, stroke caused ≈1 of every 20 deaths in the United States) (1). Its etiology is a change in blood flow to a specific area of the brain due to ischemia or hemorrhage, and it is usually manifested as brain dysfunction with consequent effects such as hemiparesis, dysphasia, ataxia, diplopia, or visual field loss. Strokes are diagnosed by physical and neurological examination, with the help of neurological scales specifically developed to quantify the impairment caused by a stroke, in particular the NIH Stroke Scale (NIHSS). This scale originally consisted of a 15-item examination (2), then amended to an 11-item examination (3), scored on a scale from 0 to 2, 3, or 4 depending on the item, for a total score ranging from 0 (normal function) to 42 (severe stroke). Several studies have reported that the baseline NIHSS (taken at hospitalization/diagnosis time) is a good predictor of outcome after a stroke (4–7). Diagnostic tools for strokes include CT scans (with or without contrast), MRI scans (especially diffusion-weighted imaging – DWI, and with magnetic resonance angiography – MRA), Doppler ultrasound, and digital subtraction angiography. In particular, for ischemic stroke, MRI scans have shown a higher sensitivity and specificity than CT scans without contrast (8). Once patients are hospitalized, electroencephalograms (EEG) are used to continuously monitor their brain function as well as to drive clinical management, since EEG abnormalities are typical manifestation of an ischemic stroke. In particular, quantitative electroencephalogram (qEEG) (9) has been used for monitoring and formulating prognosis in acute and sub-acute ischemic stroke (10). Of all the numerical parameters that can be obtained from the qEEG, of particular interest are the ratio of mean scalp delta to alpha power [known as the alpha delta ratio (ADR), or its inverse the delta alpha ratio (DAR)] (11, 12), the power ratio index (PRI) of mean “slow” (delta and theta) to mean “fast” (alpha and beta) activity (12–14), and the brain symmetry index (BSI or mBSI) (15, 16).

Standard treatment plans for patients affected by ischemic stroke involve fibrinolytic therapy (administration of recombinant tissue-type plasminogen activator – rt-PA), antiplatelet agents (such as aspirin), and mechanical thrombectomy (removal of the clot causing the blood flow obstruction). After the acute phase is concluded, the most effective rehabilitation programs involve carefully directed, well-focused, repetitive practice to relearn skills that are lost when part of the brain is damaged.

Saccades are fast eye movements that allow humans to voluntarily very quickly change the direction of gaze. Extensive studies have been conducted to characterize the different brain and eye mechanisms generating such movements and how different pathologies affect them (17). A number of standard parameters have been used to characterize saccades: latency or reaction time (the time it takes for the eyes to start moving once a stimulus is presented), velocity (at how many deg/s the eyes move), amplitude (how many degrees the eyes move), and duration (how much time it takes) (18). All of these eye movements can be quantified with diagnostic equipment, such as video-nystagmography (VNG), but they can be observed at the bedside as well. Standardized objective examination of eye movements is of great value in the detection and clarification of sub-clinical lesions in the central nervous system. Even patients with multiple sclerosis (MS) with lesions beyond the primary visual pathway have both saccadic latency and smooth pursuit abnormalities of oculomotor dysfunction (19). Patients suffering from mild closed-head injury also demonstrate prolonged saccadic latencies, and quantitative tests of oculomotor function may provide sensitive markers of cerebral dysfunction (20) that can assist and direct patient assessment. For instance, a cerebral vascular lesion in the right and/or left hemisphere produces a general slowing in the saccadic latency and a general reduction in the accuracy of saccades with respect to a healthy subject’s performance (21). Abnormalities in the control of saccades have been described in patients with cerebral pathology (22), suggesting that they might be robust biomarkers that could be utilized in guiding and interpreting treatment outcomes. Discrepancy in horizontal and vertical tilt angle coefficients can cause eye positions to lie on a twisted rather than a planar surface, resulting in eye velocities that change during a visual saccade (23). The coordination of eye movements is dependent upon the non-linear addition of visual saccades and the pursuit components of catch-up saccades that can be measured to assess function and disability (24). There are many variables that can result in different clinical scenarios for patients with similar disease states or injuries. For example, elderly patients demonstrate an increased latency and decreased peak velocity from age-related degenerative changes in the central nervous system with diseases of the central nervous system often causing saccadic disorders (25). Different disease states and sites of neurological injury may affect one component of a visual task while not affecting another. Alzheimer’s patients show increased latency to initiation of saccades but no difference in their amplitude and velocity when compared to healthy controls (26). We have observed slowing of visual saccades and saccadic intrusions of visual pursuits in patients with acute middle cerebral artery (MCA) infarction. Abnormal saccadic intrusions consisting of frequent sporadic horizontal square wave jerks occur in a large percentage of patients with acute or chronic focal cerebral lesions (27). Low-amplitude cerebral square wave jerks can be detected clinically by fundoscopy at the bedside. Reflexive visually guided saccade triggering may be facilitated or inhibited by the cerebral cortex. Pierrot-Deseilligny and colleagues observed pathology of saccades made toward and away from suddenly appearing visual targets in patients with limited unilateral cerebral infarction (28). Different phenomenology of eye movements have been observed with lesions of both the right and left cerebral hemisphers. For example, ischemic lesions of the left frontal eye field (FEF) have been associated with abnormal reflexive visually guided saccades (gap and overlap tasks), antisaccades, predictive saccades, memory-guided saccades, smooth pursuit, and optokinetic nystagmus (29). Eye-movement analysis not only identifies functional lesions but can also act as a biomarker for treatment outcomes. Hemispatial neglect affects the ability to explore space on the side opposite a brain lesion that is also mirrored in abnormal saccadic eye-movement patterns that provide a sensitive means to assess the extent of neglect recovery (30). Russell and colleagues provided the first evidence for a deficit in remapping visual information across saccades underlying right-hemisphere constructional apraxia (RHCA) (31). RHCA is a common disorder after right parietal stroke, often persisting after initial problems such as visuospatial neglect have resolved. Concurrent saccade programing is bilaterally impaired with extensive right cerebral damage with an inability to produce a corrective saccade within 100 ms after the end of a primary saccade (32). Visual field defects after striate lesions are associated with changes in the frontoparietal network underlying the cortical control of saccades, but may improve search strategies with appropriate training of saccades (33). Nelles and colleagues used functional magnetic resonance imaging (fMRI) to study the effects of eye-movement training (EMT) on cortical control of saccades (34). EMT induced altered brain activation in the striate and extrastriate cortex as well as in oculomotor areas and a relative decrease of activation in the left FEF. The cerebellum plays a major role in saccadic adaptation representing a well-established model of ­sensory–motor plasticity (35). The cerebellum remains intact after MCA infarction, while the intraparietal sulcus may be the neural substrate for remapping of the visual environment by saccadic training (36). But saccade training may not be enough in EMT as repetitive contralesional smooth visual pursuit training has been shown to induce superior, multimodal therapeutic effects in mild and severe chronic stroke patients with neglect syndrome (37).

Exploratory findings suggest that measurements of saccades, smooth pursuit, and vergence are useful in detecting changes associated with mild traumatic brain injuries (38), and it is reasonable to utilize them in other brain syndromes, including stroke. EMT has been used with vestibular rehabilitation in the successful treatment of Post-Traumatic Stress Disorder (PTSD) in combat veterans after traumatic brain injury (39–41). Dong and colleagues evaluated the sensitivity of measuring cognitive processing in the ocular motor system as a marker for recovery of deficit in post-stroke patients (42). They tested ocular motor function and compared outcomes in the NIHSS score, modified Rankin Scale (mRS), and standard cognitive function assessments. Ocular motor function was more sensitive in identifying cognitive dysfunction and improvement compared with NIHSS or mRS. They concluded that ocular motor assessment demonstrates cognitive effects of even mild stroke and may provide improved quantifiable measurements of cognitive recovery post-stroke. We desired to see if EMT might be beneficial in the treatment of acute MCA infarction and hypothesized that it would result in positive changes of qEEG and NIHSS.

## Materials and Methods

This study was a single-center, double-blind, randomized controlled clinical trial performed at our Institutional Hospital Intensive Care Unit and conducted in accordance with the Declaration of Helsinki with equipoise. The protocol was approved by the ethics committee of our Institution. Written informed consent was obtained from every potential participant prior to randomization. The effect of traditional stroke therapy (aspirin regimen) combined with a subject-specific EMT was investigated in subjects affected by MCA stroke, and its outcome compared with a “control” group consisting of subjects affected by the same pathology and receiving only the aspirin regimen. We utilized the DAR, PRI, and BSI calculated by qEEG, and NIHSS as outcome measures of intervention.

### Participants

Subjects were recruited from patients with acute MCA ischemic stroke admitted to our intensive care unit. Patients with a presumptive diagnosis of acute MCA ischemic stroke were screened within 48 h following stroke onset. Investigators verified eligibility and obtained written informed consent before randomization to two groups.

### Sample Size

The planned sample of 17 subjects in each treatment group was calculated to give the study 80% power to detect a 30% reduction in NIHSS at a 0.05 significance level for a two-sided test. The calculations assumed that 20% of participants would be lost to follow-up or non-compliant or would die of other causes.

### Inclusion Criteria

Non-disabling ischemic MCA stroke (mRS ≤3):

- Onset within 48 h before randomization.
- No previous history of cerebral strokes and functionally independent (mRS of 0 or 1) pre-morbidity.
- Focal neurological deficit of likely atherothrombotic origin classified as ischemic stroke by questionnaire/algorithm and confirmed as new cerebral infarction consistent with symptoms by cranial computed tomography and brain magnetic resonance imaging.
- Age >39 years.
- Agreement to participate in this study.
- Written informed consent.

### Exclusion Criteria

- A previous history of cerebral stroke.
- Potential sources of emboli (atrial fibrillation within 30 days of stroke, prosthetic cardiac valve, intracardiac thrombus or neoplasm, or valvular vegetation).
- Other major neurological illness that would obscure evaluation of recurrent stroke.
- Refractory depression, severe cognitive impairment, alcoholism or other substance abuse.
- General anesthesia or hospital stay of ≥3 days, any type of invasive cardiac instrumentation, or endarterectomy, stent placement, thrombectomy, or any other endovascular treatment of carotid artery within 30 days prior to admission to intensive care unit or scheduled to be performed.

### Randomization, Intervention, and Follow-up

**Figure 1:**
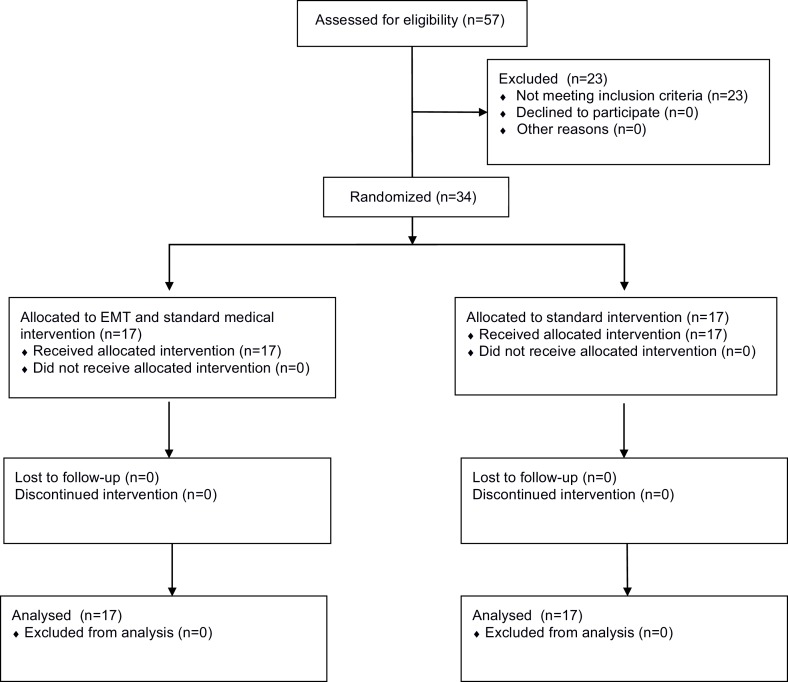
**CONSORT diagram showing the flow of participants**.

Fifty-seven subjects who had symptoms and signs of acute MCA ischemic stroke within the first 48 h of clinical evolution were admitted to our intensive care unit. Of these, 34 subjects (age 43–83 years old) met the inclusion criteria and were enrolled in this study. They had no previous history of cerebral strokes and were functionally independent (mRS of 0 or 1) pre-morbidity. The subject design was reviewed with subjects and/or their families who were offered a place in the study once informed consent was obtained. Each subject underwent a CT scan study when admitted to exclude hemorrhagic strokes, and a second CT study was performed after 72 h of stroke onset. The MCA ischemic stroke was diagnosed according to clinical history, neurological, and imaging exam. All participants consented to be admitted to the study and then were randomly assigned to “treatment” and “control” groups. The allocation of participants was programed by the statistical coordinating center, encrypted, and entered into a data entry program installed on a study computer at our institution. After computer verification that all eligibility criteria had been met, participants were treated according to their groups with both groups receiving standard medical care and support in the intensive care unit. The “control” group (11 males and 6 females, age 58 ± 10.7 years) was treated only with aspirin (125 mg/day). The “treatment” group (13 males and 4 females, age 61.9 ± 11.67 years) received the same aspirin regimen as well as subject-specific EMT. Figure 1 illustrates the CONSORT diagram showing the flow of participants.

### Treatment Group Intervention

Ipsilateral saccades are generated by the contralateral cerebral cortex and we desired to utilize extraocular muscle targets that are associated with cerebellar reflexogenic activation in the plane of the anterior and posterior canals. This strategy has been used with success in the investigation of the treatment of PTSD in combat veterans (39, 40). We prepared video targeting of the exercises on Apple iPADs using Apple Keynote software with a 2 mm red circular ball target on a blue screen background. Subjects with left MCA ischemic stroke performed diagonal saccades to appearing targets (with a fixation duration of 2 s each) using gap paradigm from the lower left to the upper right corner of the tablet monitor, followed by smooth pursuit of the target from the upper right corner to the lower left. The saccadic EMT activates the combination of right superior rectus and left inferior oblique muscles that have reflexogenic connections to the right cerebellum. Subjects with right MCA ischemic stroke performed the same type of EMT along the opposite diagonal of the display (lower right to upper left), activating the combination of left superior rectus and right inferior oblique muscles that have reflexogenic connections to the left cerebellum. This was followed by smooth pursuit of the target from the upper left corner to the lower right. Each treatment session consisted of three repetitions of the saccades/smooth pursuit sequence, and subjects received three such treatments a day. Each repetition took ~3 min with the entire intervention session taking <15 min with short breaks between repetitions.

### Intervention Both Groups

All subjects underwent EEG testing upon admission and 7 days afterward. Using a Medicid-05 (I. C. NEURONIC S.L., Zaragoza, Spain), with a gain of 20,000, sampling frequency of 200 Hz, filter band pass of 0.3–30 Hz with a “notch” filter at 60 Hz. The noise level of the EEG recording was 2 μV RMS and the recordings were performed at an environmental temperature of ~23°C. Copper electrodes coated with silver chloride were placed on the scalp at 19 monopolar derivations of the International 10/20 System with linked ear lobes as a reference. Electrode-skin impedance was <10 kΩ. Total time of EEG data collection per session was 330 s. Patients were recumbent, awake, and relaxed. For each subject, 24 artifact free segments of 2.56 s duration were visually selected by an expert electroencephalographer and used for the subsequent standard qEEG analysis [power spectrum in the delta (<4 Hz), theta (4–7 Hz), alpha (7–14 Hz), and beta (14–30 Hz) frequency bands] using the Neuronic EEG 6.0 software (I. C. NEURONIC S.L., Zaragoza, Spain). A custom script in MATLAB^®^ (The MathWorks, Inc., Natick, MA, USA) was used to calculate the DAR, the PRI, and the BSI that were used as outcome measures for all subjects. The NIHSS was also administered to all subjects upon admission and 7 days afterward.

### Statistical Analysis

The statistical analysis of the outcome measures was performed using IBM^®^ SPSS^®^ Statistics release 20.0.0 (IBM Corporation, Armonk, NY, USA) on the pre–post qEEG measures for both “treatment” and “control” groups and on the pre–post NIHSS scores. The normality of the distributions of the data was verified using Kolmogorov–Smirnov with Lilliefors Significance Correction and Shapiro–Wilk tests of normality. Since these data were found to be normally distributed, Multivariate General Linear Model (M-GLM) analysis was performed to assess the presence of differences between the two groups in the pre-treatment data, i.e., to verify if the two groups were different to begin with. The existence of a difference in pre–post changes between the “treatment” and “control” groups was investigated by performing a Multivariate Repeated Measures General Linear Model (M-RM-GLM), with repeated measures being the pre and post measures and the factor being the treatment modality. The same M-RM-GLM was performed separately on the two groups to verify if the two different treatment modalities were able to produce statistically significant changes in the outcome measures.

## Results

**Figure 2:**
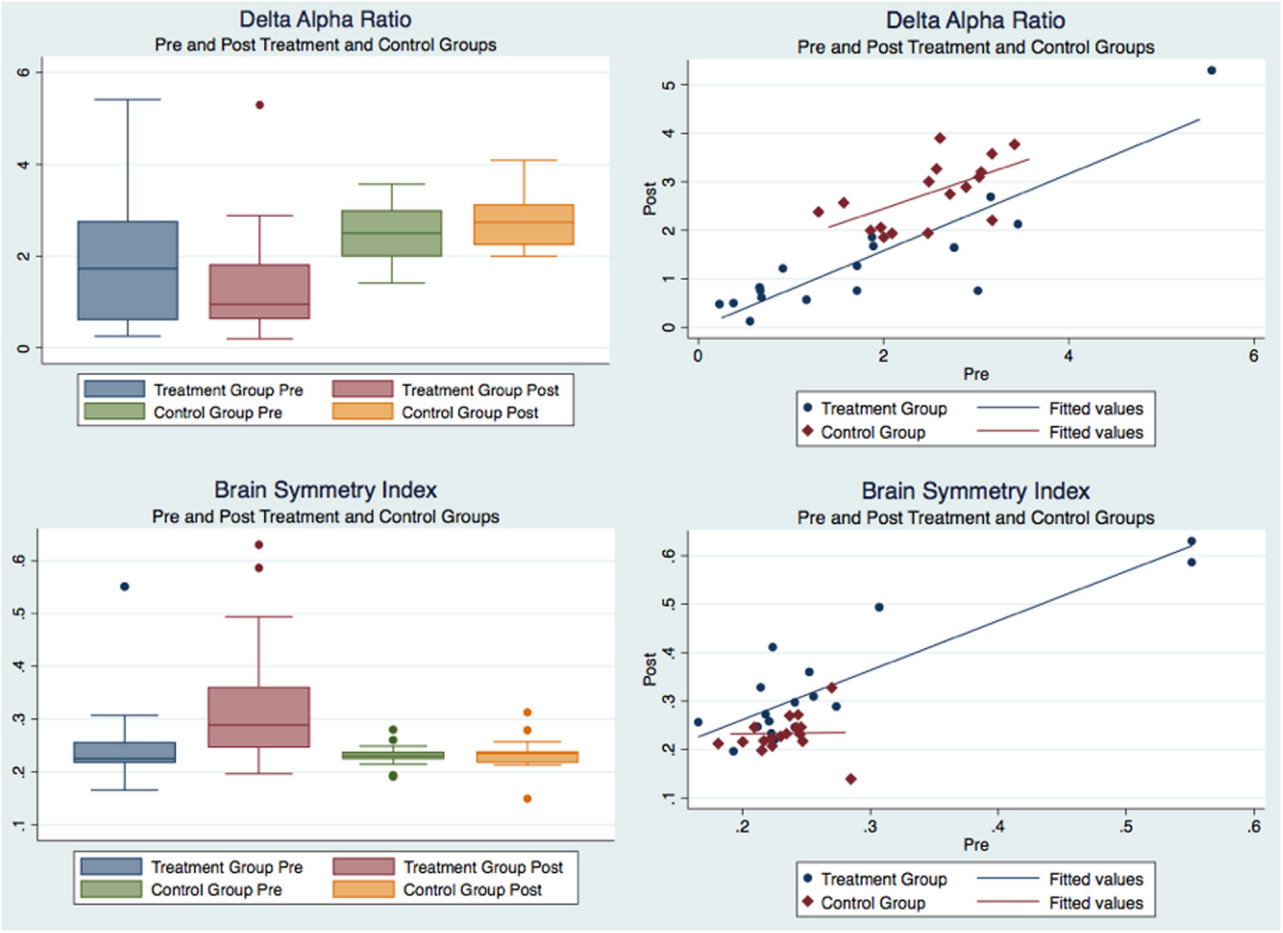
**Pre and post delta–alpha ratio and brain symmetry index of “treatment” and “control” groups**.

**Figure 3:**
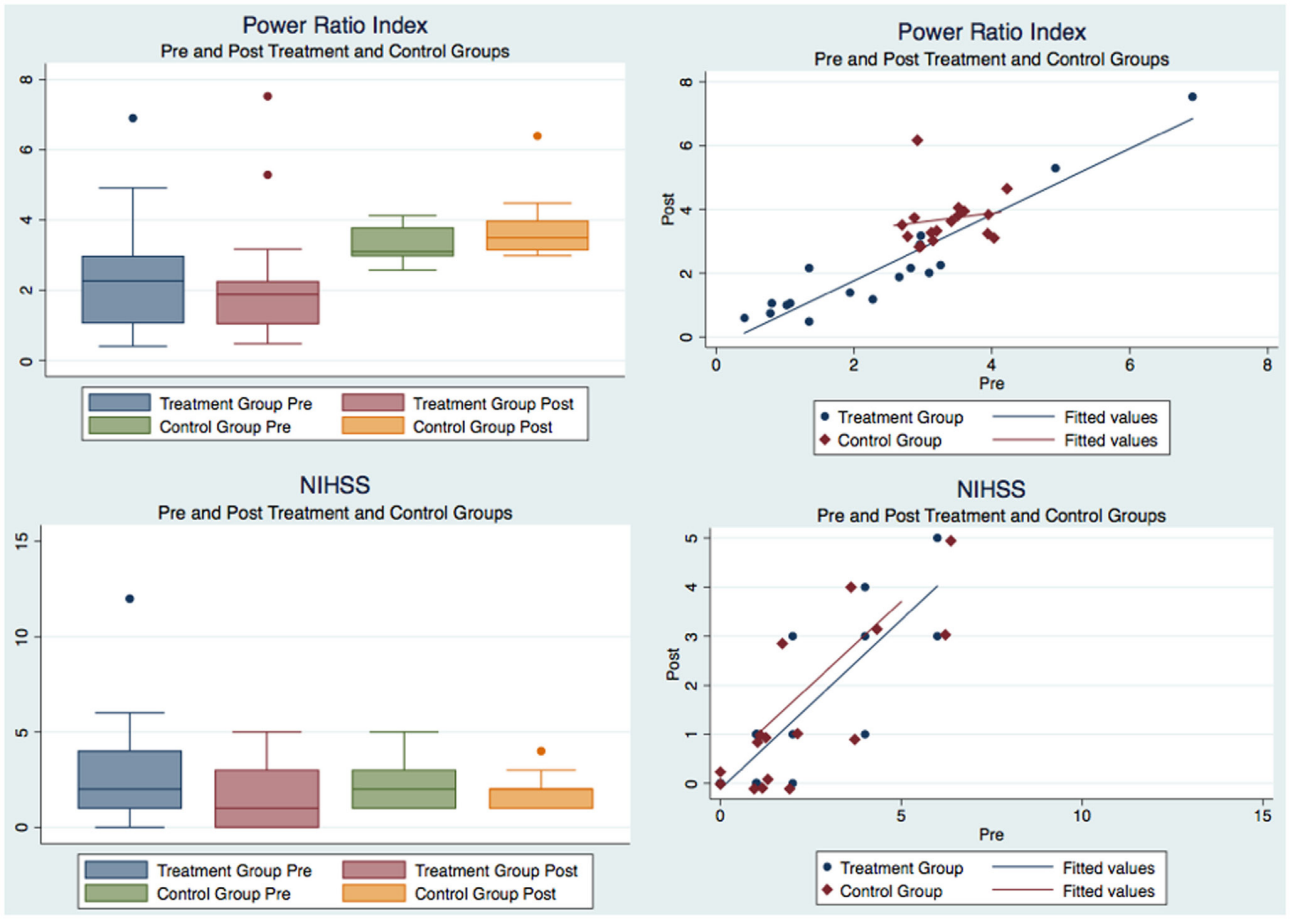
**Pre and post power ratio index and NIHSS scores of “treatment” and “control” groups**.

**Figure 4:**
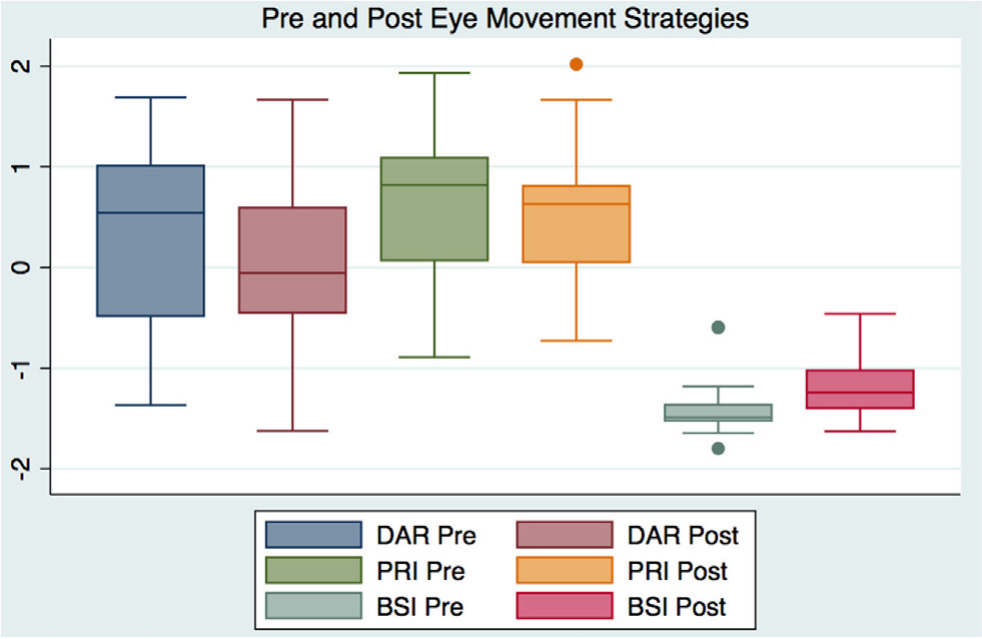
**Box plot comparing pre and post delta–alpha ratio, power ratio index, and brain symmetry index for the “treatment” group**.

The descriptive statistics for the pre and post outcome measures as well as for their paired pre–post changes for the “treatment” and “control” groups are reported in Table 1. Table 2 reports the results of the statistical analyses performed on the data: to quantify the presence of statistically significant differences between the two groups in the pre-treatment data, and in the pre–post results between and within groups. Their significance (*p* value) and effect size (calculated as partial eta squared) are also reported in the same table. A partial eta squared of 0.02 is considered a small effect, 0.13 a medium effect and 0.26 a large effect. Figure 2 depicts the pre and post DAR and BSI of “treatment” and “control” groups. Figure 3 depicts the pre and post PRI and NIHSS scores of “treatment” and “control” groups. Figure 4 illustrates using box plots the changes pre and post in the DAR, PRI, and BSI for the “treatment” group.

**Table 1:** **Mean, its 95% confidence interval (CI), and Standard deviation (SD) of the Pre, Post, and Pre–Post Change for the NIHSS, DAR, PRI, and BSI measures of “treatment” (subjects receiving EMT therapy in conjunction with the standard aspirin regimen) and “control” (subjects receiving only the standard aspirin regiment) groups**.

Measure	Group	Pre		Post		Pre–post change	
		Mean (CI)	SD	Mean (CI)	SD	Mean (CI)	SD
NIHSS	Treatment	2.82 (1.38: 4.26)	3.03	1.44 (0.67: 2.21)	1.63	−0.81 (−1.34: −0.28)	1.11
	Control	2.29 (1.66: 2.92)	1.33	1.86 (1.41: 2.31)	0.95	−0.43 (−0.67: −0.18)	0.51
DAR	Treatment	1.77 (1.10: 2.44)	1.40	1.40 (0.81: 1.99)	1.24	−0.37 (−0.67: −0.0.07)	0.63
	Control	2.48 (2.19: 2.77)	0.60	2.76 (2.46: 3.06)	0.64	0.28 (0.02: 0.54)	0.55
PRI	Treatment	2.39 (1.60: 3.18)	1.66	2.17 (1.30: 3.04)	1.82	−0.22 (−0.51: 0.07)	0.61
	Control	3.32 (3.09: 3.55)	0.48	3.69 (3.30: 4.08)	0.81	0.37 (−0.04: 0.78)	0.87
BSI	Treatment	0.27 (0.22: 0.32)	0.11	0.33 (0.27: 0.39)	0.13	0.06 (0.03: 0.09)	0.06
	Control	0.23 (0.22: 0.24)	0.02	0.23 (0.22: 0.24)	0.03	0.00 (−0.02: 0.02)	0.04

**Table 2:** **Results of the statistical analyses performed on the data, including the question under examination, each considered parameter, its significance (*p* value) and the effect size (calculated as partial eta squared)**.

Statistical question	Measure	Significance (*p* value)	Effect size (partial eta squared)
Are the two groups significantly different pre-treatment?	Multivariate	0.305	0.164
	NIHSS	^#^	^#^
	DAR	^#^	^#^
	PRI	^#^	^#^
	BSI	^#^	^#^
Are the pre–post treatment changes significantly different between the two groups?	Multivariate	0.004***	0.402
	NIHSS	0.162	0.066
	DAR	0.003***	0.243
	PRI	0.029	0.141
	BSI	0.001***	0.279
Are the pre–post treatment changes in the “control” group significant?	Multivariate	0.011*	0.699
	NIHSS	0.008**	0.429
	DAR	0.055	0.212
	PRI	0.098	0.162
	BSI	0.774	0.005
Are the pre–post treatment changes in the “treatment” group significant?	Multivariate	0.008**	0.631
	NIHSS	0.037*	0.243
	DAR	0.026*	0.272
	PRI	0.158	0.121
	BSI	0.000***	0.550

*^#^Value not calculated because multivariate *p* did not reached required statistical significance (*p* < 0.05)*.

*Statistical significance *p* < 0.05.

**Statistical significance *p* < 0.01.

***Statistical significance *p* < 0.005.

*Bold font means significant values*.

### Patient Follow-up Data

We have had no patient follow-up data on this preliminary study, but have scheduled all subjects for follow-up with repeat diagnostics at 1 year and at yearly times after the initial long-term follow-up. We will report our outcomes to long-term follow-up when they are available.

### Efficacy of Treatment

The M-GLM analysis on the initial measurements (“pre”) of the outcome measurements (first question in Table 2) indicated that the “treatment” and “control” groups are not different to begin with, with an overall multivariate tests significance of *p* = 0.305 (observed power = 0.341). After verifying the sphericity of the data using Mauchly’s test of sphericity, the M-RM-GLM analysis on the pre/post measures with the group as a factor (second question in Table 2, confirmed by Figures 2 and 3) showed that the differences in the changes between the two groups are indeed statistically significant. Specifically, the multivariate tests showed that the changes are different overall with a *p* = 0.004 and observed power of 0.922 and the tests of between-subjects effects and parameter estimates showed that the changes in the DAR, PRI, and BSI are different between the “treatment” and “control” groups with *p* of 0.003, 0.029, and 0.001 and observed power of 0.875, 0.602, and 0.926 respectively, whereas the NIHSS change is not significantly different between the two groups (*p* = 0.162). The M-RM-GLM analysis on the pre and post measures of the “control” group (third question in Table 2, confirmed by Figures 2 and 3) showed that there is a statistically significant difference in the pre and post measurements for this group (*p* = 0.011 with observed power of 0.879), but this difference is produced mostly by the change in NIHSS, which is the only measure changing significantly with *p* = 0.008 and observed power of 0.823. The same analysis on the “treatment” group (fourth question in Table 2, confirmed by Figure 4) showed that the pre/post measures are statistically significantly different for this group: the multivariate tests show that the measures are different overall with a *p* = 0.008 and observed power of 0.882 and the univariate tests and the tests of within-subjects contrasts show that NIHSS, DAR, and BSI are different with *p* of 0.037, 0.026, and 0.000 and an observed power of 0.568, 0.633, and 0.985 respectively, whereas the PRI is not different to a statistical significance (*p* = 0.158, observed power of 0.286).

## Discussion

We did not find any published studies that investigated outcome measures in the treatment of acute ischemic stroke using the NIHSS and electrical brain activity after EMT. Our results show that the group of stroke patients undergoing EMT, although not initially different from the “control” group, had a significant improvement of the electrical brain activity as measured by the DAR and BSI qEEG indices. Such improvement was not observed for the “control” group. Furthermore, the improvements in all the qEEG indices considered, i.e., DAR, PRI, and BSI, were significantly larger in the patients treated with EMT than in the controls. We did not investigate a functional relationship between qEEG findings in this pilot study, but other investigators have considered qEEG as a biomarker for neurological function. Song and colleagues (43) concluded that qEEG measures of background rhythm frequency (BRF) and relative power in the qEEG theta band are potential predictive biomarkers for cognitive impairment in patients with cerebral infarcts. These biomarkers may be valuable in the early prediction of cognitive impairment in patients with cerebral infarcts. Our findings suggest that EMT might change the qEEG and have the potential to decrease cognitive impairment in MCA ischemic stroke patients. Song and colleagues (43) also demonstrated that the risk hazard of developing cognitive impairment was 14 times higher for those with low BRF than for those with high BRF (*p* < 0.001). We have found that EMT increases BRF and perhaps decreases the risk hazard of developing cognitive impairment. Schleiger and colleagues (44) also analyzed correlations between post-stroke qEEG indices and cognition-specific functional outcome measures. They reported highly significant correlations with cognitive outcomes: frontal DAR (ρ = −0.664, *p* ≤ 0.001) and global, relative alpha power (ρ = 0.67, *p* ≤ 0.001). We have demonstrated that EMT changes these qEEG indices and as a consequence may have a functional effect specific to cognition-specific outcomes and clinical decision-making. Other investigators have utilized electrophysiological measurements to identify the potential therapeutic effects of various treatments in acute stroke. For example, Liao and colleagues (45) utilized electrophysiology to evaluate neural and vascular responses of the rat cortex to peripheral sensory stimulation following ischemic insult. They demonstrated neural recovery and the preservation of neurovascular function as well as an optimal time window of treatment that might result in minimal infarct volume in the ischemic hemisphere. Our findings of qEEG changes after EMT have led us to postulate that EMT might also be associated with neural recovery and better functional outcomes. The DAR has also been correlated with motor function recovery. Zhang and colleagues (46) evaluated the temporal alterations of neural activities using EEG from the acute phase to the chronic phase, and compared EEG with the degree of post-stroke motor function recovery in a rat model of focal ischemic stroke. The DAR was found to have the highest correlation coefficients with the motor function recovery. The statistically and substantively significant qEEG changes that we have reported after EMT would suggest that our therapy might be of use in the treatment and rehabilitation of motor function. Our study was specific to observe whether EMT would result in changes of qEEG and NIHSS without measuring other functional neurological changes. Other investigators have used similar technology to explore the relationship between qEEG global indexes and their association with functional outcome after neurorehabilitation in stroke patients. Leon-Carrion and colleagues (47) found that qEEG indexes and other clinical variables were correlated with functional recovery after neurorehabilitation. They suggested that the ratio between delta and alpha may play a significant role in predicting and monitoring functional rehabilitation outcome. We agree, and our findings that EMT changes the DAR suggest a functional application in the treatment of stroke along with other neurorehabilitation tools. We have demonstrated statistically significant changes in the NIHSS after EMT. The NIHSS offers a reliable approach to capture the true response patterns that are associated with function, outcome, and mortality post-stroke (48).

The addition of simple EMT to a patient’s treatment paradigm has demonstrated statistically significant changes in outcome measures and is a low cost, safe, and effective complement to standard treatment in MCA ischemic stroke. These results complement previous studies utilizing EMT discussed in the introduction to this report.

### Limitations

The outcome measures include only the three qEEG parameters and the NIHSS. The NIHSS is a scale of stroke severity and does not provide any insight as to functional changes. The study would have benefited from the inclusion of some functional outcome related to the rationale, e.g., change in visual tracking, cognitive and functional testing, etc. Other investigators have found that the outcomes we have utilized have been associated with functional changes in neurological function. We expect that EMT will also be associated with functional changes and improvement of outcomes after stroke treatment. We intend to address functional outcome measurements in a new randomized controlled study as our present investigation is considered a pilot from which to guide and direct future investigations and did not include other functional measurements.

## Author Contributions

FC: designed the study and the eye-movement strategies, wrote the manuscript, and contributed to the statistical analysis. EO: contributed to the study design, reviewed and edited the manuscript, and contributed to the statistical analysis. GP: contributed to the study design, reviewed and edited the manuscript, and contributed to the statistical analysis. CW: reviewed and edited the manuscript and contributed to the statistical analysis. CM: prepared IRB submissions, patient recruitment, and review of the manuscript. GE: coordinated subject diagnosis and treatment, and reviewed the manuscript. AP: reviewed and edited the manuscript and contributed to subject assignment. JC: reviewed the manuscript and contributed to the data collection and compilation. CB: reviewed the manuscript and contributed to subject treatment assignments.

## Conflict of Interest Statement

The authors declare that the research was conducted in the absence of any commercial or financial relationships that could be construed as a potential conflict of interest.
